# Antipsychotic Medication and QT Prolongation

**DOI:** 10.12669/pjms.315.8998

**Published:** 2015

**Authors:** Paramdip Singh Chohan, Raman Mittal, Afzal Javed

**Affiliations:** 1 Dr. Paramdip Singh Chohan, MBChB, DCH. Manor Court Avenue, Nuneaton, Warwickshire, CV11 5HX, UK; 2 Dr. Raman Mittal, MRCPsych. Manor Court Avenue, Nuneaton, Warwickshire, CV11 5HX, UK; 3 Dr. Afzal Javed, FRCPsych, M. Phil. Manor Court Avenue, Nuneaton, Warwickshire, CV11 5HX, UK

**Keywords:** Antipsychotics, QT Prolongation, Sudden Cardiac Death

## Abstract

The QT interval represents ventricular depolarisation and repolarisation. Prolongation of this interval can lead to life-threatening complications. These can include arrhythmias such as Torsades de Pointes and Ventricular Fibrillation, which may ultimately lead to death. Many risk factors have been identified in prolonging the QT interval, one of which is medication commonly used in the treatment of Psychiatric ailments. This article describes Antipsychotic drugs causing prolonged QT interval and the possible underlying mechanisms alongside the current best practice on the management of this potentially fatal complication.

## What is the QT interval?

**Fig.1:**
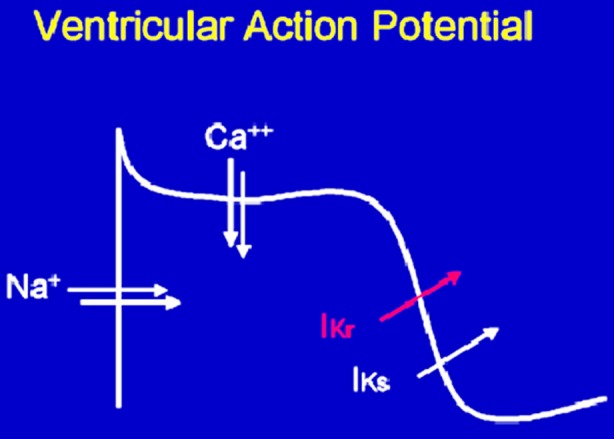
The Ventricular Action Potential.^2^

The QT interval, measured as the interval between the initiation of the Q wave and the termination of the T wave on an Electrocardiogram (ECG), is a measure of ventricular depolarisation and repolarisation.^1^ Due to its variation with heart rate the QTc offers a more indicative value. A number of formulas including Bazzet’s formula and Freidreich’s formula can be used to calculate the QTc.^1^ Normal values differ between males and females. Prolonged QTc is defined as >450msecs in males and >470msecs in females.^2^ In order to understand factors which could influence the QTc it is important to have some biochemical knowledge of the ventricular action potential of the cardiac myocytes. This involves four phases as depicted in Fig.1 below. Phase zero represents initial repolarisation with influx of Sodium. Phase one represents early repolarisation with inactivation of the Sodium currents and onset of transient outward Potassium currents. A balance between outwards Potassium currents and inward Calcium currents accounts for phase 2, the plateau phase. Rapid repolarisation (Phase 3) follows when Calcium currents are inactivated whilst Potassium currents remain open. Finally, Phase 4 represents the resting membrane potential.^1^

## Why is it important?

Prolongation of the QT interval can lead to fatal consequences. These are mainly the result of Sudden Cardiac Death (SCD) caused by arrhythmias such as Torsades de Pointes. The mortality associated with this arrhythmia is estimated to be around ten percent.^2^ Hence, it is important for Physicians to have knowledge of risk factors which could prolong the QTc. These can include, cardiac conditions such as Congenital long QT syndrome and Hypertension. Systemic diseases such as Hypothyroidism, Liver disease and Renal disease are also indicated as causative factors. Further risk factors may include electrolyte imbalances, female gender and drugs.^4^ Drug causes include: antiarrhythmics, antibiotics, antiemetics, antidepressants and antipsychotics.^5^

## What is its relevance to Psychiatry?

It is well known that patients with an enduring mental health condition such as Schizophrenia have a reduced life expectancy of >20 years. Cardiovascular Disease accounts for the highest number of natural deaths.^6^ Psychotropic medication can lead to a number of cardiovascular complications, one of which is prolongation of the QT interval. The mechanism for such medication to prolong the QT interval is likely to be due to their effect on Potassium channels and the human ether-a-go-related (hERG) gene.^1^ As Potassium channels may remain open for a longer duration, ventricular repolarisation and consequently the QT interval are prolonged. Up to 90 percent of patients who develop Tosades de Pointes with concurrent use of non antiarrhythmic medication, including Psychotropic medication, have been shown to have a QTc >500msec.^2^ This further demonstrates the link between prolongation of QTc and this potentially fatal arrhythmia. Of the commonly used antidepressants, Citalopram and Escitalopram have been found to prolong the QT interval.^6^ Beach et al.^1^ showed that Thioridazine, Ziprazidone and IV Haloperidol were the antipsychotics which had the greatest propensity for prolonging the QT interval and to be associated with Torsades de Pointes. Aripiprazole was shown not to have a positive effect on either of these outcomes.^1^ Ozeki et al.^7^ concluded that Chlorpromazine, Levomepromazine and IV Haloperidol all statistically significantly increase the QT interval. It has been widely suggested that second generation antipsychotics have a lower tendency to prolong the QT interval when compared to first generation antipsychotics.^7^

## Our Experience

We recently carried out an audit of patients currently under our service. Our service provides specialised care to patients in the community with a history of a current or past Psychosis related illnesses. Out of a random sample of one hundred audited patients we found that five patients had a prolonged QTc interval on their most recent ECG. Table-I summarises the details for these patients.

**Table-I:** Summary of patients found to have prolonged QTc on ECG.

	Age	Sex (M/F)	Diagnosis	Current Medication	Past Medical History	QTc (msec)
Case A	52	M	Schizophrenia	Clozapine 325mg – Daily dose	Valvular Heart Disease	458
				Citalopram 20mg OD	Dyslipidaemia	
Case B	33	M	Schizophrenia	Clozapine 400mg - Daily dose	Epilepsy	470
				Pregablin 100mg BD		
				Epilim 700mg BD		
Case C	46	M	Schizophrenia/Schizoaffective Disorder	Clozapine 475mg - Daily dose	Diabetes	457
				Diazepam 5mg BD	Dyslipidaemia	
				Paroxetine 30mg ON	Asthma	
				Depakote 500mg BD		
				Benzhexol 5mg OD		
				Propranolol 160mg OD		
				Omeprazole 20mg OD		
				Metformin/Sitagliptin 50mg OD		
				Atorvastatin 40mg ON		
				Salbutamol PRN		
Case D	35	M	Schizophrenia in Remission	Clopixol Depot 400mg - monthly	Nil	464
				Procyclidine 5mg OD		
Case E	43	M	Schizophrenia	Clozapine 800mg – Daily dose	Diabetes	461
				Citalopram 40mg OD	Anaemia	
				Pregablin 100mg BD	Hypothyroidism	
				Metformin 500mg OD		
				Simvastatin 80mg ON		
				Lisinopril 2.5mg OD		
				Lansoprazole 15mg OD		
				Levothyroxine 50 micrograms OD		

Findings of this audit show that all five patients (with a prolonged QTc) were on medication which could prolong their QT interval. Some of the patients also had Physical Health conditions which could predispose them to having a prolonged QTc. For instance, Case E was a known case of Hypothyroidism. Our findings are consistent with current literature. The majority of our patients were taking Clozapine, studies implicating Clozapine as a cause of prolonging QTc are sparse. Further research into this area is needed.

## What is the current best practice?

Prolonged QT is the most common cause of marketed drug withdrawal. Monitoring and managing patients on Psychiatric medication for prolonged QT interval is essential in order to reduce the risk of life-threatening complications.^6^ It is important to take a thorough clinical history from all patients, which should include a family history of CVD, Arrhythmias and SCD. Furthermore, a baseline ECG followed by annual ECGs is advised to monitor patients on such medication. Unless the QTc is >500msec ruling out other causes for QT prolongation before discontinuing Psychotropic medication is recommended, as this in itself may lead to detrimental effects. If the QTc is >500msec then medication will need to be discontinued and advise sought from a Cardiologist. The use of Psychotropic medication as monotherapy where possible is strongly suggested, if further therapies are indicated then avoiding the concomitant use of two medications known to prolong the QT interval is recommended.^3^

In conclusion, it is evident that a number of medications used for the treatment of Psychiatric conditions (especially Antipsychotic medication) may be associated with QT prolongation and worsening cardiovascular outcomes. The identification of patients who are more susceptible to this complication is imperative. Active monitoring of patients with regular ECGs is therefore essential. It must be remembered that antipsychotic medication is only one of a vast number of risk factors for prolonging the QT interval. Hence, other causes should be excluded prior to stopping medication, unless the QTc is >500msecs. The importance of conducting a careful risk-benefit analysis is integral. Although treatment with Psychotropic medication such as antipsychotics is associated with an increased risk for Sudden Cardiac Death, overall mortality remains lower than for patients who are left untreated.
